# YOLOX-Ray: An Efficient Attention-Based Single-Staged Object Detector Tailored for Industrial Inspections

**DOI:** 10.3390/s23104681

**Published:** 2023-05-11

**Authors:** António Raimundo, João Pedro Pavia, Pedro Sebastião, Octavian Postolache

**Affiliations:** 1 Instituto de Telecomunicações (IT), Av. Rovisco Pais, 1, 1049-001 Lisboa, Portugal; aslro@iscte.pt (A.R.); joao.pedro.pavia@ulusofona.pt (J.P.P.); pedro.sebastiao@iscte-iul.pt (P.S.); 2 Department of Information Science and Technology, Iscte—Instituto Universitário de Lisboa, Av. das Forças Armadas, 1649-026 Lisboa, Portugal; 3 COPELABS, Universidade Lusófona, Campo Grande 376, 1749-024 Lisboa, Portugal

**Keywords:** industrial inspections, computer vision, deep learning, object detection, YOLOX-Ray, attention mechanisms, loss function

## Abstract

Industrial inspection is crucial for maintaining quality and safety in industrial processes. Deep learning models have recently demonstrated promising results in such tasks. This paper proposes YOLOX-Ray, an efficient new deep learning architecture tailored for industrial inspection. YOLOX-Ray is based on the You Only Look Once (YOLO) object detection algorithms and integrates the SimAM attention mechanism for improved feature extraction in the Feature Pyramid Network (FPN) and Path Aggregation Network (PAN). Moreover, it also employs the Alpha-*IoU* cost function for enhanced small-scale object detection. YOLOX-Ray’s performance was assessed in three case studies: hotspot detection, infrastructure crack detection and corrosion detection. The architecture outperforms all other configurations, achieving $mAP_{50}$ values of 89%, 99.6% and 87.7%, respectively. For the most challenging metric, $mAP_{50:95}$, the achieved values were 44.7%, 66.1% and 51.8%, respectively. A comparative analysis demonstrated the importance of combining the SimAM attention mechanism with Alpha-*IoU* loss function for optimal performance. In conclusion, YOLOX-Ray’s ability to detect and to locate multi-scale objects in industrial environments presents new opportunities for effective, efficient and sustainable inspection processes across various industries, revolutionizing the field of industrial inspections.

## 1. Introduction

Industrial maintenance inspection is a critical task for ensuring the efficient and safe operation of industrial facilities. The inspection process entails diagnosing, inspecting and repairing equipment and machinery to prevent breakdowns and extend their operational lifetime. However, traditional inspection methods are becoming increasingly ineffective and time-consuming as the complexity and automation of industrial systems is increasing. Because of this, innovative solutions are required to improve the efficiency and effectiveness of industrial inspection [1].

In recent years, Computer Vision (CV) has become increasingly important for industrial applications. It has been identified as a key technology for increasing productivity, lowering costs and improving safety in a variety of industries. In industry, CV is used to extract relevant information from visual data using cameras, sensors, Machine Learning (ML) and Deep Learning (DL) algorithms. These data can then be used for decision making, quality control, predictive maintenance and other industrial applications [2].

With the rapid advancements in Artificial Intelligence (AI) and CV, DL models have emerged as a viable alternative to manual inspection methods. These models can analyze large amounts of visual data quickly and accurately, allowing the detection and localization of defects, anomalies and other problems with more precision and consistency than human inspectors. In recent years, DL models have demonstrated promising results in various industrial inspection tasks, such as defect detection, anomaly localization and quality control [3].

DL models employ object detection and classification techniques to identify anomalies and defects in infrastructure and various industrial equipment. By analyzing images or video streams, these techniques can pinpoint and locate specific features or flaws in the equipment or infrastructure. These models are trained utilizing extensive datasets of labeled images. Object detection constitutes a fundamental challenge in computer vision, with the primary objective of identifying objects of interest within images or videos [4].

In fact, several object detectors have been proposed in the literature in recent years, such as YOLO [5], SSD [6], RetinaNet [7], Faster R-CNN [8], Mask R-CNN [9] and Cascade R-CNN [10]. Moreover, significant progress has been made in computer vision and object detection over the years, with DL models achieving State-of-the-Art (SoTA) results on benchmark datasets. However, these models are frequently evaluated in controlled settings using high-quality, well-annotated images that may not accurately represent real-world conditions.

SoTA object detection models are not specifically designed for industrial inspection tasks and their performance in complex, real-world scenarios may be sub-optimal. In manufacturing environments, for example, DL models may struggle to detect minor defects on surfaces or locate objects that are partially hidden by other items. Similarly, in warehouse settings, these models may struggle to identify objects that are partially hidden or at different distances from the camera. Such challenges highlight the importance of an object detection architecture that excels at accurately identifying and locating objects in industrial inspection tasks, regardless of the complexities inherent in real-world situations.

The You Only Look Once (YOLO) object detector family is a well-known group of single-stage DL models that enable real-time object detection in images and videos. The YOLO detector family has evolved over time, with different iterations achieving SoTA performance on object detection benchmarks [11]. Despite the improvements in YOLO versions and other object detectors, they are based only on CNN traditional architectures. Although CNNs can extract relevant information from input data, their ability to selectively focus on the most important information is often limited [12].

Attention Mechanisms are a fundamental component of DL models, particularly for tasks such as natural language processing and CV. Attention mechanisms are designed to help models selectively focus on relevant parts of input data, allowing them to learn important patterns and features in a more efficient manner [13]. In CV, attention mechanisms have shown significant improvements in tasks such as object detection, image classification and image segmentation. By selectively attending to parts of an image, attention mechanisms can help models focus on relevant features, such as object boundaries or salient regions and ignore irrelevant information, such as background noise or occlusions. This can lead to improved accuracy and faster training times [13,14].

Recent advances in attention mechanisms have also resulted in the creation of novel attention modules, such as the Simple Parameter-free Attention Module (SimAM), a lightweight and efficient attention mechanism that can be easily incorporated into existing DL architectures. Such attention mechanisms have been shown to significantly improve the performance of object detection models, particularly for small object detection and multi-scale object detection, both of which are critical for industrial inspection applications [15].

In this paper, we propose the ‘YOLOX-Ray’, a novel DL architecture that is built upon the YOLO family of detectors and designed specifically for industrial maintenance tasks. The main contributions of this paper are as follows:

1. We introduce the SimAM attention mechanism into the YOLOX’s backbone. This enables a better feature extraction and feature fusion on the architecture’s neck;
2. The proposed architecture implements a novel loss function, Alpha-*IoU*, which enables better bounding-box regression for small object detection.

The remaining sections of this paper are structured as follows: Section 2 provides a review of related work within the topic under study. The proposed method is detailed in Section 3. Section 4 presents the case studies, experimental tests, results and analysis of the ablation study. Finally, conclusions are outlined in Section 5.

## 2. Related Work

### 2.1. YOLO

In recent years, the You Only Look Once (YOLO) [5] family of single-stage object detectors has received considerable attention within the CV domain due to its remarkable accuracy and real-time performance. The ability of YOLO to process images in real time makes it an excellent choice for industrial tasks that require fast and efficient anomaly detection.

The YOLO family of algorithms has a significant advantage in terms of detection speed, which makes it well-suited for real-time object detection applications. However, there are some limitations, such as lower accuracy when compared to two-stage detectors such as Faster R-CNN. Depending on the problem, reduced accuracy does not always imply poor performance. Achieving a balance between inference time, complexity and accuracy values is critical in order to select the best algorithm for specific use cases [16].

The YOLO family includes both official and unofficial versions. The official versions are those published by the original authors, such as YOLOv1 [5], YOLOv2 [17] and YOLOv3 [18]. Unofficial versions, on the other hand, are those created by various authors and adapted from official versions, such as YOLOv5 [19], YOLOR [20] and YOLOX [21].

### 2.2. YOLOX

The YOLO family of object detectors has achieved considerable popularity due to its rapid inference speed and high accuracy. Nevertheless, researchers continue to push the boundaries of object detection by introducing novel techniques and architectures. One such innovation is the YOLOX detector, as presented in the paper ‘YOLOX: Exceeding YOLO Series in 2021’ by Ge et al. [21].

YOLOX’s anchor-free design is a key feature. Traditional object detectors, such as YOLOv4 and YOLOv5, predict object locations using anchor boxes. YOLOX, on the other hand, uses a center-based approach to directly localize objects using centers or key points [22], rather than relying on predefined boxes or anchors. This anchor-free approach is simpler, more flexible and more intuitive than anchor-based methods, which require many hyperparameters and increase computational demands [23,24].

Another notable feature of YOLOX is its decoupled head. The prediction head is tightly coupled with the feature extractor in earlier YOLO versions (YOLOv3 through YOLOv5) and other traditional object detectors, making performance improvement difficult through head modifications. By employing a 1 × 1 convolutional layer for each level of the Feature Pyramid Network (FPN), reducing the feature channel to 256 and then adding two parallel branches with two 3 × 3 convolutional layers each for classification and regression tasks, YOLOX’s design allows for improved feature extraction and performance. This design allows for greater flexibility in head architecture and, as a result, improved performance [21,22].

**Figure 1:**
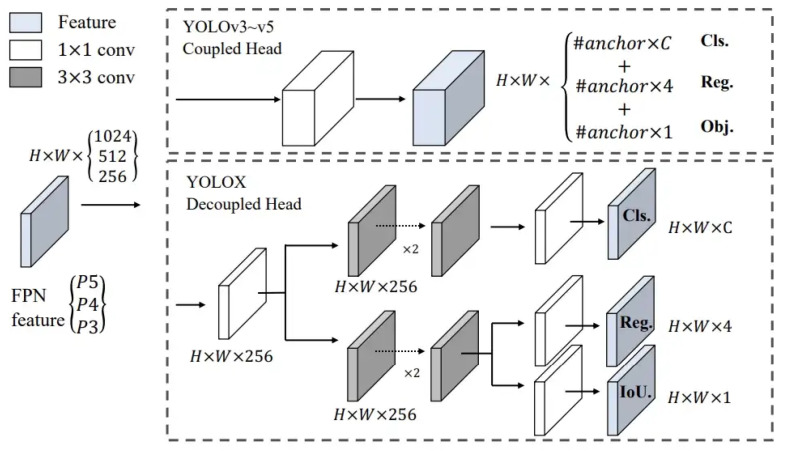
Coupled head vs. Decoupled head (Source: [21]).

Figure 1 illustrates the difference between a coupled head of YOLOv3-v5 and the decoupled head used in YOLOX.

YOLOX also introduces a novel label assignment strategy called Simplified Optimal Transport Assignment (SimOTA). This strategy involves selecting the *k* most confident predictions from the current model and using the Optimal Transport Assignment (OTA) [25] algorithm to identify the *k*-best matching Ground-Truth (*GT*) boxes. The chosen predictions and ground-truth boxes are then employed to train the model and the process is repeated for each batch of data. This approach results in an increase in Mean Average Precision (mAP) [25].

Lastly, YOLOX incorporates advanced augmentations, such as MixUP [26] and MOSAIC [27], to further improve performance. MixUP is an image augmentation technique that blends two images, while MOSAIC combines multiple images into one, assisting the network in detecting smaller objects. These techniques enable the model to better generalize to unseen data [21,23].

### 2.3. Attention Mechanisms

In the field of DL, attention mechanisms have emerged as a powerful technique, particularly in CV and natural language processing tasks. These mechanisms allow models to selectively focus on relevant features or regions within input data, while minimizing the impact of less important information. Attention mechanisms boost a model’s ability to learn complex patterns and dependencies in data by dynamically weighting and aggregating information based on its relevance [28].

In Section 2.3.1, Section 2.3.2, Section 2.3.3 and Section 2.3.4, we discuss, respectively, some types of attention mechanisms: Squeeze-and-Excitation Networks (SENet), Convolutional Block Attention Module (CBAM), Coordinate-Attention (CA) and the Simple Parameter-free Attention Module (SimAM). Such mechanisms were presented to highlight the main differences between them as candidates to be implemented in our architecture.

#### 2.3.1. SENet

Squeeze-and-Excitation Networks (SENet) [29] represent a significant advancement in the field of DL for CV tasks. The core concept of SENet is the introduction of a self-attention module known as the Squeeze-and-Excitation (SE) block, which aims to recalibrate channel-wise feature responses by explicitly modeling channel interdependencies. The SE block is divided into two stages: the squeeze operation, which aggregates global spatial information by using global average pooling and the excitation operation, which generates channel-wise weights by using a fully connected layer with a sigmoid activation function. These weights are then applied to the original feature maps in order to highlight the most relevant channels while suppressing the least informative ones. SENet significantly improves performance in various CV tasks such as image classification and object detection by incorporating the SE block into existing Deep Neural Networks (DNNs) [29].

#### 2.3.2. CBAM

Convolutional Block Attention Module (CBAM) [30] is an influential development in the realm of DL, particularly for CV tasks. CBAM is a lightweight, generic attention module designed to enhance the representational power of CNNs by incorporating both spatial and channel attention mechanisms. The CBAM module sequentially processes the feature maps generated by a convolutional layer, first applying the Channel Attention Mechanism (CAM) to recalibrate channel-wise feature responses, followed by the Spatial Attention Mechanism (SAM) to highlight the most salient regions in the feature maps. The channel attention component employs global average pooling and global max pooling operations to generate channel-wise descriptors, while the spatial attention component uses 1D convolutions to capture spatial dependencies. By integrating the CBAM module into existing CNN architectures, it effectively refines the feature representations, resulting in improved performance in various CV tasks [30].

#### 2.3.3. Coordinate-Attention

The Coordinate-Attention (CA) module is a relatively recent attention [31] mechanism designed to improve the ability of deep learning models to capture essential spatial information and dependencies within the input data. It specifically addresses the limitations of traditional attention mechanisms that only focus on channel-wise or spatial dependencies, ignoring the relationships between the spatial positions of features. The CA module works by explicitly incorporating coordinate information into the attention mechanism, allowing the model to capture both spatial and channel-wise dependencies more effectively [31].

The CA module consists of two primary components: the Coordinate-Channel Attention (CCA) and the Coordinate-Spatial Attention (CSA). The CCA focuses on capturing channel-wise dependencies by considering the coordinate information of each feature in the channel, while the CSA captures spatial dependencies by taking into account the relationships between the spatial positions of features. By combining these two components, the CA module provides a more comprehensive understanding of the input data, ultimately leading to improved performance in various computer vision tasks [31].

#### 2.3.4. SimAM

The Simple, Parameter-Free Attention Module (SimAM) is a novel attention mechanism proposed by Yang et al. [15] that aims to provide a more lightweight and efficient approach to feature refinement. Unlike the traditional attention mechanisms, which require additional training layers and parameters, SimAM is designed to be both simple, parameter-free and computationally efficient, making it suitable for resource-limited real-time object detection applications.

The key idea behind SimAM is to efficiently produce true 3D weights that operate on both channel and spatial domains, inspired by the coexistence of feature-based and spatial-based attention in the human brain. The authors argue that the calculation of 3D weights should be straightforward and lightweight, allowing the module to be efficiently integrated into DL architectures [15].

**Figure 2:**
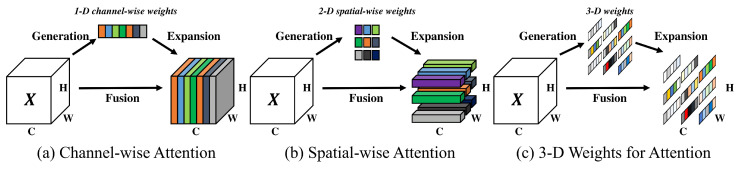
CAM and SAM attention mechanism vs. SimAM (Source: [15]).

Figure 2 illustrates the difference between the SimAM, SAM and CAM attention mechanisms.

The authors’ approach was inspired by visual neuroscience to estimate the importance of individual neurons based on a feature map. Informative neurons have distinct firing patterns from neighboring neurons and exhibit spatial suppression effects. The most basic method for locating these neurons is to measure the linear separability of one target neuron from other neurons. The authors proposed an energy function for each neuron that has a minimum value when the target neuron can be linearly separated from all other neurons in the same channel [15].

The energy function for each neuron can be calculated using Equation (1).

(1) $$e_{t}\left(w_{t},b_{t},x_{i},y\right)={\left(y_{t}-\hat{t}\right)}^{2}+\frac{1}{M-1}\sum_{i=1}^{M-1}{\left(y_{o}-{\hat{x}}_{i}\right)}^{2}$$

In the energy function, the linear transforms of *t* and $x_{i}$ are given by $\hat{t}=w_{t}t+b_{t}$ and ${\hat{x}}_{i}=x_{i}w_{t}+b_{t}$, respectively. The transform’s weight and bias are symbolized by $w_{t}$ and $b_{t}$, respectively. *t* represents the target neuron; $x_{i}$ denotes other neurons in the same channel; *M* is the total number of neurons on that channel and $y_{t}$ and $y_{o}$ are two different values (binary labels) [15].

The energy function can be minimized using a closed-form solution, which significantly reduces computation costs by calculating the mean and variance over all neurons and reusing them for all neurons on that channel. By doing so, the importance of each neuron can be determined based on the energy function’s minimal value. The lower the energy, the more distinctive and important the neuron is for visual processing [15].

With respect to $w_{t}$ and $b_{t}$, the final energy function can be obtained by using Equation (2).

(2) $$e_{t}\left(w_{t},b_{t},x_{i},y\right)=\frac{1}{N-1}\sum_{i=1}^{N-1}{\left(-1-\left(x_{i}w_{t}+b_{t}\right)\right)}^{2}+{\left(1-\left(w_{t}t+b_{t}\right)\right)}^{2}+\lambda w_{t}^{2}$$

The closed-form solution can be calculated using Equations (3) and (4).

(3) $$w_{t}=-\frac{2(t-\mu_{t})}{{\left(t-\mu_{t}\right)}^{2}+2\sigma_{t}^{2}+2\lambda }$$

(4) $$b_{t}=-\frac{1}{2}\left(t+\mu_{t}\right)w_{t}$$

The minimal energy can then be computed by using Equation (5).

(5) $$e_{t}^{min}=\frac{4(\sigma^{2}+\lambda )}{{\left(t-\mu \right)}^{2}+2\sigma^{2}+2\lambda }$$

where *t* is the target neuron; $x_{i}$ represents other neurons in the same channel; *N* is the total number of neurons on that channel, $\mu_{t}$ and $\sigma_{t}^{2}$ are the mean and variance calculated over all neurons except *t* in that channel; μ and $\sigma^{2}$ are the mean and variance calculated over all neurons in that channel. The regularization parameter is denoted by λ.

SimAM employs this energy function to generate an attention map that reflects the significance of each neuron. The resulting attention map can then be integrated into any feature extraction component of a DL architecture (such as the backbone). SimAM is a computationally efficient module that is easily adaptable to various DL architectures [15].

Incorporating the SimAM attention mechanism into the YOLOX-Ray architecture significantly improves its object detection capabilities. The YOLOX-Ray architecture, as presented in Section 3, can effectively refine features in both the channel and spatial domains by introducing this novel attention module, resulting in a more accurate and robust object detection performance. The SimAM module is lightweight and efficient, with no additional training layers or parameters required, making it a more computationally efficient solution. Furthermore, the attention mechanism is modeled after human visual processing, in which feature-based and spatial-based attention coexist, allowing for more natural and effective information selection during detection tasks. By incorporating the SimAM module, the YOLOX-Ray architecture becomes better equipped to handle complex and challenging real-world scenarios, such as those found in industrial inspections and anomaly detection tasks, ultimately improving the model’s performance and practical applicability.

### 2.4. Loss Functions

Loss functions, which quantify the difference between the model’s predictions and the *GT*, are critical in the training process of DL models. Models learn to make more accurate predictions by optimizing the loss function during the training process, improving their overall performance. In object detection, recognizing an object is regarded as a classification task, while localizing it within a rectangular bounding box is treated as a regression task. Bounding boxes are typically represented by the coordinates of their upper-left and lower-right corners. Object detectors are trained using a multi-task loss function that accounts for both classification and regression tasks. This loss function evaluates the discrepancy between the predicted and *GT* bounding boxes, generating gradients for back-propagation to update the network parameters [32].

The general equation to calculate the loss function for object detection tasks is given by Equation (6),

(6) $$L_{total}=L_{cls}\left(P,\;P_{GT}\right)+\beta L_{reg}\left(B,\;B_{GT}\right)$$

By analyzing Equation (6), the loss function consists of two components: the classification loss function, denoted as $L_{cls}$ and the regression loss function, denoted as $L_{reg}$. Typically, the Cross-Entropy (CE) loss function is used as the classification loss function, measuring the discrepancy between the predicted class probability, *P*, and the *GT* class probability, $P_{GT}$. In contrast, the regression loss function evaluates the difference between the predicted bounding box, B=(x,y,h,w) and the GT bounding box, $B_{GT}=\left(x_{GT},y_{GT},h_{GT},w_{GT}\right)$. The weight of $L_{reg}$ can be adjusted by a hyperparameter β [32].

However, this loss function is not explicitly designed to align with its evaluation metric, the Intersection-over-Union (*IoU*), as it has been shown to be sensitive to multi-scale bounding boxes [33,34].

#### 2.4.1. IoU Loss

The Intersection-over-Union (*IoU*) evaluation metric is a popular choice in object detection tasks, as it measures the degree of overlap between the predicted and GT bounding boxes. Given a predicted bounding box $B_{p}$ and a GT bounding box $B_{GT}$, the *IoU* score can be calculated by using Equation (7):

(7) $$IoU=\frac{B_{p}\cap B_{GT}}{B_{p}\cup B_{GT}}$$

IoU values range from 0 to 1, with higher values indicating better overlap between the bounding boxes. The IoU loss function is defined as demonstrated by Equation (8):

(8) $$L_{IoU}=1-IoU$$

The IoU loss is used during training to encourage the model to produce more accurate bounding box predictions [35]. It has been demonstrated to be effective in various object detection architectures, as it directly optimizes the evaluation metric of interest. However, the IoU loss may suffer from gradient vanishing issues, particularly when there is a significant misalignment between the predicted and GT bounding boxes [33]. To address this limitation, several variants of the IoU loss have been proposed, such as the Generalized-*IoU* (GIoU) [35], Distance-*IoU* (DIoU) [33] and Complete-*IoU* (CIoU) [33] loss functions, which aim to improve the learning process by incorporating additional geometric information or distance metrics.

#### 2.4.2. Generalized-IoU

The Generalized-*IoU* (GIoU) loss function is an extension of the traditional IoU metric, aiming to better capture the overlap between two bounding boxes.

The GIoU loss can be calculated by using Equation (9):

(9) $$L_{GIoU}=1-GIoU=1-\frac{IoU-(\frac{A_{C}-A_{U}}{A_{C}})}{IoU}$$

where $A_{C}$ is the area of the smallest enclosing box (convex hull) and $A_{U}$ is the union area of the two bounding boxes.

#### 2.4.3. Distance-IoU

The Distance-*IoU* (DIoU) loss function aims to address the issue of localization accuracy by incorporating the distance between the box centers into the calculation [33].

The DIoU loss can be calculated by using Equation (10):

(10) $$L_{DIoU}=1-DIoU=1-\left(IoU-\frac{d^{2}\left(b_{p},b_{GT}\right)}{c^{2}}\right)$$

where $b_{p}$ and $b_{GT}$ denote the box central points of $B_{p}$ and $B_{GT}$, $d^{2}$ is the squared Euclidean distance between those points and $c^{2}$ is the squared diagonal distance of the smallest enclosing box (convex hull).

#### 2.4.4. Complete-IoU

The Complete-*IoU* (CIoU) loss function, proposed by the same authors as the DIoU loss [33], further improves the localization accuracy by incorporating both the aspect ratio consistency and the center distance into the calculation.

The CIoU loss can be calculated by using Equation (11):

(11) $$L_{CIoU}=1-CIoU=1-\left(IoU-\frac{d^{2}\left(b_{p},b_{GT}\right)}{c^{2}}-v\cdot \alpha \right)$$

where $b_{p}$ and $b_{GT}$ denote the box central points of $B_{p}$ and $B_{GT}$, $d^{2}$ is the squared Euclidean distance between those points, $c^{2}$ is the squared diagonal distance of the smallest enclosing box (convex hull), *v* is the aspect ratio term and α is a trade-off parameter that balances the contribution of aspect ratio consistency in the loss function.

The aspect ratio term, *v*, is calculated by using Equation (12):

(12) $$v=\frac{4}{\pi^{2}}\cdot {\left(\arctan\left(\frac{h_{GT}}{w_{GT}}\right)-\arctan\left(\frac{h_{p}}{w_{p}}\right)\right)}^{2}$$

where $h_{GT}$ and $w_{GT}$ are the height and width of the *GT* bounding box and $h_{p}$ and $w_{p}$ are the height and width of the predicted bounding box.

The alpha parameter, α, is calculated by using Equation (13):

(13) $$\alpha =\frac{v}{(1-IoU)+v}$$

This formulation of α ensures that the aspect ratio term has a balanced influence on the overall CIoU loss.

#### 2.4.5. Alpha-IoU Loss

He et al. [36] introduced Alpha-*IoU*, a novel loss function tailored for precise bounding box regression and object detection. This innovative loss function serves as a powerful generalization of existing IoU-based losses, offering a cohesive approach to enhancing bounding box regression [36].

The paper’s authors examine various properties of the Alpha-*IoU* loss, such as order preservingness, loss and gradient re-weighting. They demonstrate that by selecting a suitable value for α (specifically, α>1), the Alpha-*IoU* loss can effectively improve bounding box regression accuracy by increasing the loss and gradient of high IoU objects.

The Alpha-*IoU* loss function is defined by Equation (14),

(14) $$L_{\alpha -IoU}=\frac{1-IoU^{\alpha }}{\alpha },\alpha >0$$

The Alpha-*IoU* loss function, $L_{\alpha -IoU}$, offers a versatile reweighting strategy by adjusting the parameter α, enabling the achievement of varying degrees of bounding box regression accuracy. This adaptability stems from the fact that α is sensitive to the target’s IoU value and its absolute and relative properties allow for deriving the majority of existing IoU losses. This re-weighting capacity leads to enhanced accuracy as assessed by Average Precision (AP) at different IoU thresholds [37]. In addition, researchers from [37,38] indicated that most *IoU*-based loss functions (such as GIoU, DIoU and others) can be derived from the Alpha-*IoU* equation.

The paper presents empirical evidence from multiple benchmark datasets and models, demonstrating that the Alpha-*IoU* loss outperforms other *IoU*-based losses. Furthermore, the authors reveal that the Alpha-*IoU* loss exhibits increased robustness for small datasets and noisy bounding boxes [36].

Table 1 depicts the experimental results achieved via the CIoU and Alpha-*IoU* loss functions on the DOTA dataset for different object scales [38].

Alpha-*IoU* outperforms commonly used *IoU*-based loss functions such as GIoU, DIoU and CIoU when it comes to detecting small objects. This happens because Alpha-*IoU* can account for scale differences between small and large objects, improving its performance in detecting minor differences in small bounding boxes. Alpha-*IoU* assesses the overlap between the predicted bounding box and GT more thoroughly by incorporating both the *IoU* and scale factors. As a result, detectors trained with Alpha-*IoU* are better at handling small objects, resulting in improved detection precision and overall performance.

## 3. Proposed Method

In this section, we present the YOLOX-Ray architecture and our two main contributions to enhance the YOLOX base architecture for improved performance in industrial inspection and anomaly detection tasks. The name YOLOX-Ray is inspired by the metaphorical concept of ‘X-ray vision’, symbolizing the algorithm’s ability to effectively ‘see through’ and detect problems that may be challenging for the human eye. This creative reference aims to emphasize the effectiveness of our proposed architecture in identifying and addressing issues within industrial environments, further enhancing inspection processes across various industries.

Firstly, we introduce a novel attention mechanism, SimAM, which is incorporated into the backbone of the YOLOX base architecture. This addition aims to improve feature extraction capabilities, enabling the model to focus on crucial regions within the input images. Finally, we replaced the actual IoU loss function with a novel IoU loss function called Alpha-IoU, specifically designed to enhance the detection capabilities for small objects. The implementation of the Alpha-IoU loss function increases the suitability of the model for various industrial inspection tasks, where the detection of minor defects or anomalies is of utmost importance.

### Network Architecture

YOLOX-Ray is a versatile object detection network, specifically tailored for efficient performance in industrial inspection tasks, making it well-suited for identifying multi-scale anomalies in complex industrial environments. The architecture is composed of a backbone, a neck structure and a decoupled head, which collectively enable the network to process complex image inputs and accurately detect objects across a range of sizes.

**Figure 3:**
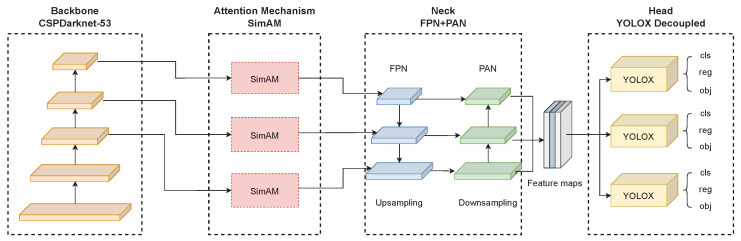
YOLOX-Ray architecture design.

Figure 3 illustrates the YOLOX-Ray architecture design.

As illustrated in Figure 3, despite the added attention mechanism, the structure follows the traditional object detector design, which is composed of the following components:

- Backbone: The backbone of the YOLOX-Ray network is based on the YOLOX base architecture, the CSPDarknet-53, which was first introduced in YOLOv4. This architecture is a modified version of the popular Darknet-53 architecture, with the addition of Cross Stage Partial (CSP) connections. Darknet-53 is a 53-layer DNN that has shown great performance on a variety of object detection tasks [39]. By combining these two structures, the CSPDarknet-53 backbone in YOLOX-Ray provides a high-level feature representation for object detection.
- Attention Mechanism: The SimAM attention mechanism, a novel mechanism that improves CNN performance by calculating attention weights in feature maps, is added at the end of the backbone without additional parameters [15]. The SimAM was added after the third layer, ‘Dark-3’, the fourth layer, ‘Dark-4’, and the fifth layer, ‘Dark-5’, of the original CSPDarknet-53 backbone, which served to improve the representation ability of feature extraction and improve feature fusion process in the neck component.
- Neck: The YOLOX-Ray neck is the same as the YOLOX base architecture, consisting of FPN and PAN structures. The neck takes the feature maps extracted by the backbone and generates a pyramid of features at different scales, allowing the network to detect objects at different scales. It performs upsampling in the Feature Pyramid Network (FPN) and downsampling in the Path Aggregation Network (PAN) [21,40].
- Head: The YOLOX-Ray architecture’s head, known as the YOLOX decoupled head, is also the same as the YOLOX base. This head is designed to perform bounding box regression and multi-class classification in parallel, allowing the network to predict the location and class of objects efficiently and effectively [21].

It is important to note that the Alpha-*IoU* loss function plays an important role in the YOLOX-Ray’s head structure. Several levels of *IoU* scores can be obtained by adjusting the α parameter in the Alpha-*IoU* loss function. By integrating this loss function into the head component, YOLOX-Ray would be able to achieve higher accuracy and efficiency for multi-scale objects.

The primary innovations and novelties of the YOLOX-Ray architecture are the integration of the SimAM attention mechanism module at the end of the backbone, as well as the use of an optimized Alpha-*IoU* loss function.

Finally, the YOLOX-Ray architecture was developed to be resilient, adaptable and efficient for detecting objects at various scales. The CSPDarknet-53 backbone, SimAM attention mechanism and optimized Alpha-*IoU* loss function ensure that the YOLOX-Ray network can detect multi-scale objects accurately and efficiently, making it an ideal solution for industrial inspection tasks and other real-time object detection applications.

In Section 4, we present the experimental tests performed on three distinct case studies, as well as an ablation study. This section also presents a performance evaluation of the YOLOX-Ray architecture, demonstrating its effectiveness in real-world scenarios. We also provide a comprehensive assessment of the impact of the SimAM attention mechanism and the Alpha-*IoU* loss function on overall performance.

## 4. Experimental Tests and Results

This section discusses the experimental tests that were performed in order to evaluate the proposed architecture’s performance in real-world industrial inspection tasks.

Given the multiple challenges and complexities inherent in industrial inspection tasks, such as changing lighting conditions, occlusions and the detection of small objects, it is critical to evaluate the proposed architecture in real-world scenarios. These experiments were carried out on three datasets representing various industrial applications to ensure that the YOLOX-Ray architecture performs effectively across a wide range of industrial inspection tasks.

The three case studies of industrial inspections are as follows:

- Case Study A: Solar Farm Thermal Inspection (available in [41]);
- Case Study B: Infrastructure Integrity Inspection (available in [42]);
- Case Study C: Bridge Cables Inspection (available in [43]).

To train and test the YOLOX-Ray architecture, a GPU-powered machine was used. The experiments were carried out on a machine equipped with the following resources:

- CPU: AMD Ryzen 7 3700X 3.6 GHz;
- GPU: 2 x NVIDIA GeForce RTX 2060TI SUPER OC 8 GB VRAM;
- RAM: 32 GB DDR4.

When compared to a CPU, using a GPU significantly accelerates DL model training and inference because GPUs are optimized for parallel computations, which are critical for the numerous operations required by DL algorithms. Furthermore, the open-source machine learning framework PyTorch was used to create the YOLOX-Ray architecture.

This section will also cover the YOLOX-Ray network hyperparameter specification, dataset structure for each case study, experimental tests and results and an ablation study to evaluate the impact of different components on overall performance.

### 4.1. Datasets Structure

The datasets for each case study were obtained from the Roboflow-100, a collection of curated multi-domain object detection datasets made available for research purposes. The Roboflow-100 datasets are diverse and cover a wide range of object detection applications, making them a popular choice among computer vision researchers and practitioners [44]. In contrast to other widely used benchmark datasets like COCO and PASCAL VOC, Roboflow-100 offers a wider variety of object classes, leading to a more flexible environment for object detection.

Furthermore, the images in the datasets were divided into three subsets, training, validation and testing, with 70% allocated to training, 20% allocated to validation and 10% allocated to testing. This division enables a more thorough evaluation of the model’s performance as well as a more precise estimation of its effectiveness on new data.

One of the main purposes of this study is to demonstrate the effectiveness and adaptability of the YOLOX-Ray architecture in several industrial inspection scenarios. Since there is no direct correlation between the proposed method and the characteristics of the dataset, the ability of the architecture to have a good perform on diverse datasets is a proof its versatility. The implementation of the Alpha-*IoU* loss function helps in multi-scale object detection [38], making the architecture suitable for detecting objects with different sizes and scales. Additionally, by resizing all images to a consistent size of 640 × 640, it is ensured that the architecture would focus on detecting relevant objects within the images while maintaining a consistent input size for each dataset.

The annotations were provided in the PASCAL VOC format, which is a widely used format for object detection annotations. Considering the dataset and the annotation format selection, the YOLOX-Ray was evaluated in a more realistic and practical context rather than in a controlled benchmark environment.

Table 2 presents the technical details of the datasets used in each case study.

**Figure 4:**
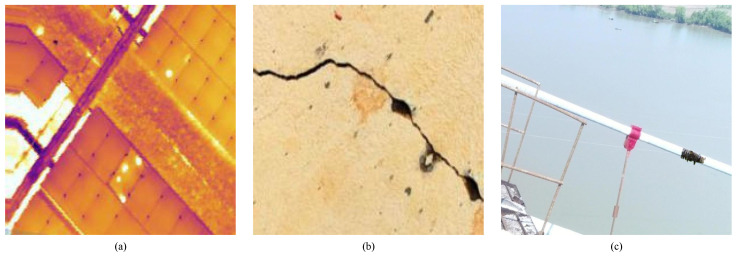
Image samples of each dataset: (**a**) Aerial thermal image of a solar farm; (**b**) Crack on a concrete infrastructure; (**c**) Corrosion on a bridge infrastructure.

Figure 4 illustrates the datasets sample images of each case study, where (a), (b) and (c) correspond to Case Studies A, B and C, respectively.

In Figure 4, image (a) serves as an example for Case Study A; image (b) represents a sample for Case Study B; and image (c) illustrates a sample from the Case Study C dataset.

### 4.2. Network Hyperparameters

The YOLOX-Ray architecture’s hyperparameters, which are essential configuration choices that can significantly impact the model’s performance, were meticulously selected to achieve optimal results.

Table 3 illustrates the network hyperparameters configured for the training process.

The data augmentation techniques used for training the YOLOX-Ray model are MOSAIC and MixUP, which are the original YOLOX architecture’s base augmentations. Hue, Saturation and Value (HSV) enhancements, as well as horizontal and vertical flip augmentations, were also included. These methods are commonly used in CV tasks to improve the model’s ability to generalize to previously unseen data [45].

The authors of the Alpha-*IoU* loss function proved that a α value of 3 produced the best results [36].

The hyperparameters were chosen based on their proven effectiveness in previous DL research and were also further optimized during the training process to ensure optimal performance for the YOLOX-Ray architecture.

In this work, the original YOLOX pre-trained models were not used as initial weights, since the usage of initial weights led to overfitting during the initial epochs of the training process. The problem of overfitting may manifest itself when it turns out that the pre-trained models are not directly related to the datasets used for such study. Consequently, to avoid this problem, we opted to train the algorithm from scratch for each case study, allowing the model to learn relevant features without being influenced by unrelated pre-existing weights.

### 4.3. Model Size

The performance of the YOLOX-Ray model was evaluated using four distinct model sizes: YOLOX-Ray-s, YOLOX-Ray-m, YOLOX-Ray-l and YOLOX-Ray-x. In CV, the depth of a DNN refers to the number of layers in the network architecture. A deeper network has more layers, which allows it to learn more complex data representations. In contrast, the network’s width refers to the number of neurons in each layer. A larger network has more neurons, allowing it to learn more detailed data information [46].

As a result, the depth and the width of the network are determined by the available computational resources, where the model will be deployed. The four models (YOLOX-Ray-s, YOLOX-Ray-m, YOLOX-Ray-l and YOLOX-Ray-x) were created by changing the network depth and width values in order to provide a set of models with different computational requirements and expected performance. The lightest and fastest model (YOLOX-Ray-s) has the lowest expected mAP values. The largest model, on the other hand (YOLOX-Ray-x), is the heaviest and slowest, but has the best expected performance in terms of mAP.

Table 4 presents the network depth and width values for each model size.

The depth and width values presented in Table 4 were derived from the model scaling techniques proposed in YOLOv5 by Ultralytics [19] and subsequently adopted in YOLOX by its authors [23]. In this work, the same logic for model scaling was applied to define the values for depth and width, considering different model sizes of the YOLOX-Ray architecture.

### 4.4. Performance Metrics

For the evaluation metrics, the *IoU* score is used as a threshold for determining whether a prediction is considered a True Positive (TP), True Negative (TN), False Positive (FP) or False Negative (FN). For example, if the *IoU* score between a predicted bounding box and the corresponding *GT* bounding box is greater than a certain threshold (e.g., 0.5), the prediction is considered a TP. On the other hand, if the *IoU* score is below the threshold, the prediction is considered an FP [47].

In this paper, the YOLOX-Ray models in terms of Precision (*P*), Recall (*R*), mAP over an IoU score of 0.5 ($mAP_{50}$), mAP on an IoU threshold of 0.5 to 0.95 ($mAP_{50:95}$)

(15) $$Precision=\frac{TP}{TP+FP}$$

(16) $$Recall=\frac{TP}{TP+FN}$$

where FN is the number of false negative detections, TP is the number of correctly predicted positive instances and FP is the number of false positive predictions.

For calculating AP [47], Equation (17) is used,

(17) $$AP=\int_{0}^{1}p\left(r\right)dr$$

Equation (18) is used for calculating mAP scores.

(18) $$mAP=\frac{1}{N}\sum_{i=1}^{N}AP_{i}$$

where *N* is the number of classes in the target dataset and $AP_{i}$ is the average precision for class *i*.

The mAP metric is widely used as a primary evaluation measure in object detection. It provides an overall evaluation of the performance of an object detection algorithm by incorporating precision and recall information. The mAP metric is used to compare the performance of various algorithms on well-known benchmark datasets such as COCO and PASCAL VOC. This metric has been widely adopted as a standard for comparing different object detection algorithms and it has been featured in numerous research publications [47].

### 4.5. Experimental Results

The YOLOX-Ray architecture’s experimental tests were carried out on three distinct case studies, as previously outlined in the present section. Consequently, the performance of the YOLOX-Ray architecture was evaluated across four different model sizes. These various model sizes were analyzed to find the optimal trade-off between performance and computational efficiency.

Conducting experimental results for different model sizes in different use cases (Case studies A, B and C) is essential for assessing the YOLOX-Ray architecture in a variety of real-world situations since different use cases present unique challenges and requirements. The YOLOX-Ray architecture must be resilient, robust and effective in detecting anomalies of varying sizes and shapes in different environments, which can only be achieved through testing on a range of use cases.

Furthermore, this section includes a comparison of image predictions (object detection) for each case study and every YOLOX-Ray model size. These images display the YOLOX-Ray architecture’s capacity to detect and to localize objects within images. The object detection scores are presented as bounding boxes surrounding each detected object, with the scores indicating the confidence level that the object belongs to the identified class. The images illustrate the YOLOX-Ray architecture’s performance in various industrial inspection use cases and with different model sizes.

The images were selected from the test subset of each case study and they represent only a single example prediction. Other predictions were made, but only the presented ones were chosen to emphasize certain strengths, limitations and differences of the YOLOX-Ray models.

**Figure 5:**
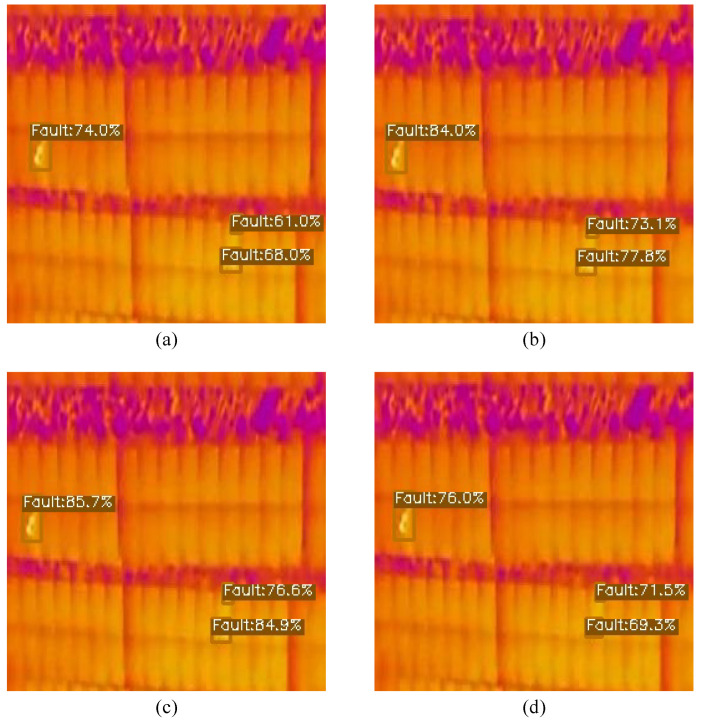
Image predictions for Case Study A: (**a**) Prediction on YOLOX-Ray-s; (**b**) Prediction on YOLOX-Ray-m; (**c**) Prediction on YOLOX-Ray-l; (**d**) Prediction on YOLOX-Ray-x.

**Figure 6:**
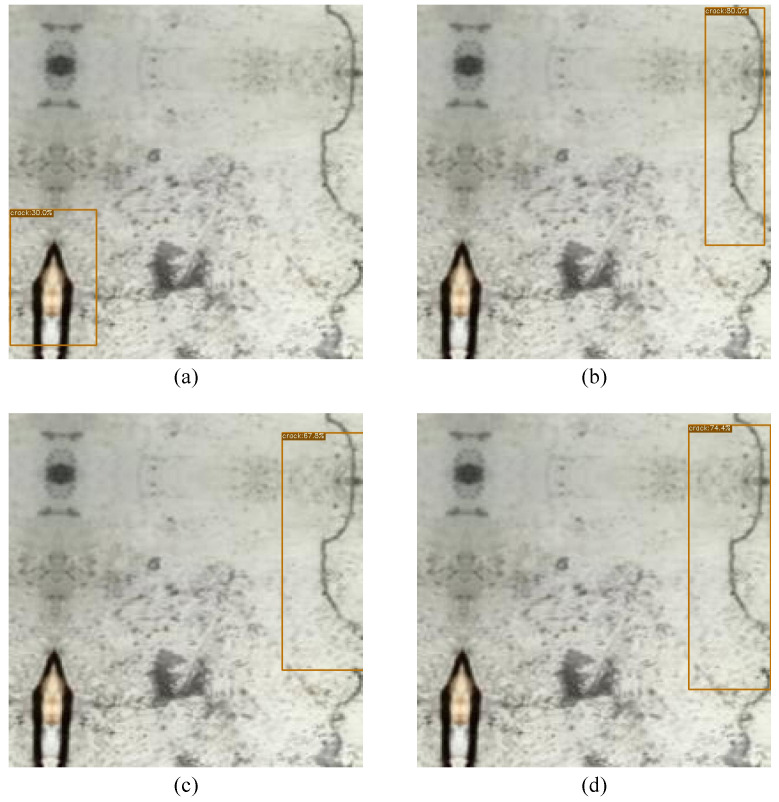
Image predictions for Case Study B: (**a**) Prediction on YOLOX-Ray-s; (**b**) Prediction on YOLOX-Ray-m; (**c**) Prediction on YOLOX-Ray-l; (**d**) Prediction on YOLOX-Ray-x.

**Figure 7:**
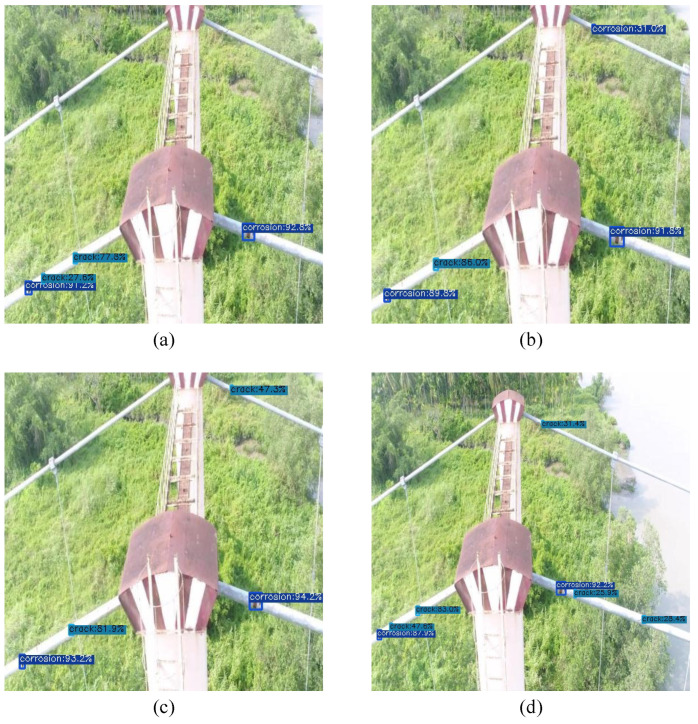
Image predictions for Case Study C: (**a**) Prediction on YOLOX-Ray-s; (**b**) Prediction on YOLOX-Ray-m; (**c**) Prediction on YOLOX-Ray-l; (**d**) Prediction on YOLOX-Ray-x.

Figure 5, Figure 6 and Figure 7 have four images, (a), (b), (c) and (d), which are the same image but with different detection scores, each for a different model. Image (a) illustrates the detection scores for the smallest model, YOLOX-Ray-s, while image (b) depicts the medium-sized model, YOLOX-Ray-m. Image (c) depicts the large model, YOLOX-Ray-l and image (d) illustrates the detection scores for the extra-large model, YOLOX-Ray-x.The evaluation results are presented in Table 5, Table 6 and Table 7, each showing the performance of the YOLOX-Ray models in terms of *P*, *R*, mAP over an IoU score of 0.5 ($mAP_{50}$), mAP on an IoU threshold of 0.5 to 0.95 ($mAP_{50:95}$), inference times in ms (Inf.) and the number of parameters in millions (Params).

### 4.6. Case Study A: Experimental Results and Predictions

Table 5 demonstrates the evaluation metrics and their values for Case Study A.

Examining Table 5 and beginning with the *P* metric, the YOLOX-Ray-m and YOLOX-Ray-l models achieved higher values (0.829 and 0.806, respectively) compared to the small model (0.73). This indicates that the medium and large models are more accurate in detecting hotspots. Curiously, the extra-large model had one of the lowest *p* values (0.733).

In terms of *R*, all models achieved high values, with YOLOX-Ray-s reaching the highest at 0.917 and YOLOX-Ray-x obtaining the lowest at 0.879. This suggests that the models were successful in identifying most anomalies present in the images, regardless of their size.

In terms of $mAP_{50}$, YOLOX-Ray-l performed the best with a value of 0.89, followed by YOLOX-Ray-s with 0.877. YOLOX-Ray-m and YOLOX-Ray-x achieved similar scores (0.872 and 0.845, respectively), with the extra-large model having the lowest score. This implies that larger models may not be optimal for this specific use case.

Regarding $mAP_{50:95}$, YOLOX-Ray-l achieved the highest score of 0.427, closely followed by YOLOX-Ray-s and YOLOX-Ray-m with 0.422 and 0.426, respectively. YOLOX-Ray-x obtained the lowest score of 0.376. This metric indicates that YOLOX-Ray-l and YOLOX-Ray-s are the most accurate models in detecting hotspots with a high IoU score.

Inference time is a crucial factor in real-time object detection applications. In this instance, YOLOX-Ray-s had the fastest inference time at 11.95 ms, followed by YOLOX-Ray-m (19.55 ms), YOLOX-Ray-l (29.22 ms) and YOLOX-Ray-x (46.56 ms). As expected, this suggests that smaller models are more efficient regarding inference time, making them better suited for real-time object detection.

Finally, the number of parameters for each model varied significantly. YOLOX-Ray-s had the fewest parameters with 8.94 million, followed by YOLOX-Ray-m with 25.28 million, YOLOX-Ray-l with 54.15 million and YOLOX-Ray-x with 99 million. This indicates that smaller models are more lightweight and may be more appropriate for resource-limited environments.

As expected, the inference time and number of parameters for each model also increased as the model’s size grew. Overall, the YOLOX-Ray architecture demonstrated solid performance in this case study, which allows for potential further improvement if the model size is not a concern.

Figure 5 illustrates the instance predictions of different models for Case Study A.

By analyzing Figure 5, it can be concluded that this image contains only small instances of the class ‘Fault’, allowing the evaluation of the YOLOX-Ray architecture’s capacity to detect small objects.

It is noticeable that the YOLOX-Ray-s model had the lowest prediction scores for all detections. In contrast, in line with the results obtained and presented in Table 5, the medium and large models achieved the best detection scores, with YOLOX-Ray-m achieving the highest prediction scores. Interestingly, the YOLOX-Ray-x model did not perform well in this specific example, illustrating that even models designed to excel can underperform compared to lighter models in certain situations.

In summary, this example demonstrates the effectiveness of the YOLOX-Ray architecture in detecting small objects and emphasizes the significance of choosing the suitable model size based on the task requirements and dataset characteristics.

### 4.7. Case Study B: Experimental Results and Predictions

Table 6 demonstrates the evaluation metrics and their values for Case Study B.

Table 6 shows the YOLOX-Ray-s model surpassed all other models in all metrics, except for a slightly lower value of $mAP_{50:95}$ when compared to the medium version (0.66 vs. 0.661). This indicates that the smaller model is adequate for achieving high performance in the crack detection task and implies that a smaller model can be a more effective solution in terms of both inference time and model complexity.

In terms of *P*, YOLOX-Ray-s also outperformed the other models, achieving a value of 0.984. The medium and large versions had slightly lower values of 0.972 and 0.962, respectively, while the extra-large version achieved a *p* value of 0.972.

All models secured high *R* values, ranging between 0.971 and 0.987. The small model achieved the highest value of 0.987, followed by the large version with a value of 0.979.

Regarding $mAP_{50}$ and $mAP_{50:95}$, all models secured high values, spanning from 0.977 to 0.996 for $mAP_{50}$ and 0.625 to 0.661 for $mAP_{50:95}$. The YOLOX-Ray-s model achieved the highest values for $mAP_{50}$, while the medium version achieved the highest value for $mAP_{50:95}$.

In terms of inference times, it is worth noting that YOLOX-Ray-s reached the lowest inference time, with a value of 9.62 ms, followed by YOLOX-Ray-m and YOLOX-Ray-l at 17.09 and 25.96 ms, respectively. As expected, the YOLOX-Ray-x model had the highest inference time, with a value of 42.53 ms.

Finally, it is important to highlight that the number of parameters remained the same across all models, since they were trained using the same configuration. The only difference was the dataset that was used for training and evaluation.

To conclude, the YOLOX-Ray-s model demonstrated the best overall performance in the crack detection task, outperforming the larger and more complex models in terms of both $mAP_{50}$ and inference times. These results suggest that smaller models can be a feasible solution for this industrial inspection task, particularly when efficiency is the key.

Figure 6 illustrates the instance predictions of different models for Case Study B.

By analyzing Figure 6, it is possible to conclude that the YOLOX-Ray-s model produced a false positive detection, which is a significant observation, suggesting that smaller models might be more susceptible to false positives. The medium model (YOLOX-Ray-m) achieved the highest prediction score of 80%, followed closely by the YOLOX-Ray-x at 74.4%.

Additionally, the YOLOX-Ray-m achieved a more accurate bounding box regression aligned with the GT box compared to other models, implying that the YOLOX-Ray-m model is better at fitting the ‘crack’ instance.

These results show that, while all models performed almost identically in terms of evaluation metrics (as shown in Table 6), false positives can still occur in weaker models. This emphasizes the importance of selecting a suitable model for each use case scenario, as well as the need to investigate additional methods for reducing false positives in smaller models.

### 4.8. Case Study C: Experimental Results and Predictions

Table 7 demonstrates the evaluation metrics and their values for Case Study C.

Table 7 displays the experimental outcomes of the YOLOX-Ray models trained and evaluated on Case Study C, which is more challenging than the other two case studies due to the presence of three classes: ‘slippage’, ‘corrosion’ and ‘crack’.

First, considering the *P* metric, the YOLOX-Ray-x model achieved the highest value of 0.832, indicating that it made fewer false positive predictions compared to other models. The YOLOX-Ray-m and YOLOX-Ray-l models also demonstrated high *p* values, at 0.829 and 0.792, respectively. However, the YOLOX-Ray-s model had the lowest *p* at 0.762, signifying a higher rate of false positives.

Next, examining *R*, which evaluates the model’s ability to accurately identify positive instances, the YOLOX-Ray-l model obtained the highest value of 0.883. The YOLOX-Ray-m and YOLOX-Ray-x models also performed well in *R*, with values of 0.878 and 0.876, respectively. The YOLOX-Ray-s model had the lowest *R* at 0.866.

Regarding $mAP_{50}$, the YOLOX-Ray-x model reached the highest value of 0.877. The YOLOX-Ray-m and YOLOX-Ray-l models also posted high $mAP_{50}$ values, at 0.871 and 0.873, respectively. However, the YOLOX-Ray-s model had the lowest $mAP_{50}$ at 0.859, indicating a lower average *P* across all thresholds.

Lastly, for $mAP_{50:95}$, representing the mean average *P* with a threshold range of 0.50 to 0.95, the YOLOX-Ray-x model achieved the highest value of 0.518. The YOLOX-Ray-l model also had a relatively high $mAP_{50:95}$ value of 0.505. The YOLOX-Ray-m and YOLOX-Ray-s models recorded values of 0.499 and 0.484, respectively.

In terms of inference times, the YOLOX-Ray-s model had the shortest time at 18.04 ms, while the YOLOX-Ray-x model had the longest time at 58.12 ms. This is expected, as larger models require more computation time.

Figure 7 illustrates the instance predictions of different models for Case Study C.

Examining Figure 7, it is evident that this figure includes multiple instances of the ‘crack’ and ‘corrosion’ classes, which the YOLOX-Ray models were expected to accurately detect.

By analyzing image (a), it is possible to conclude that the YOLOX-Ray-s model missed three ‘crack’ instances in the image, indicating room for enhancement in its detection abilities.

The YOLOX-Ray-m model’s performance was slightly inferior to that of the YOLOX-Ray-s model, as it misidentified a ‘crack’ instance as a ‘corrosion’ instance.

The YOLOX-Ray-l model achieved better prediction scores than the YOLOX-Ray-s and YOLOX-Ray-m models but still failed to identify three ‘crack’ instances in the image.

On the other hand, the YOLOX-Ray-x model, despite having lower prediction scores, successfully detected all instances in the image, making it the only model to achieve 100% object detection for this specific image.

This example underlines the variations in detection capabilities among the YOLOX-Ray models and the trade-offs between prediction scores and detection performance. Although the YOLOX-Ray-x model achieved perfect object detection, its prediction scores were lower than those of the YOLOX-Ray-l model.

Moreover, the YOLOX-Ray-s and YOLOX-Ray-m models had lower prediction scores than the YOLOX-Ray-l model but missed certain instances, signifying the necessity for model enhancements.

Overall, this example shows the importance of striking a balance between prediction scores and detection performance in object detection models and the need for ongoing research and development to improve model capabilities.

### 4.9. Ablation Study

Ablation studies play a crucial role in DL experiments as they help to determine the contributions of specific techniques, features or components added to a DL base architecture to enhance its overall performance [48].

The objective of this study is to compare the YOLOX-Ray results across all case studies when the SimAM attention mechanism is added to the YOLOX base architecture, the Alpha-*IoU* loss function is implemented and finally the YOLOX-Ray architecture, which is a combination of SimAM and Alpha-*IoU*.

Experiments were conducted using the smallest model (YOLOX-s) in all case studies, with the evaluated metrics being *P*, *R*, $mAP_{50}$, $mAP_{50:95}$, inference time in ms (Inf.) and Frames Per Second (FPS). The ablation study results can be visualized in Table 8, Table 9 and Table 10.

Incorporating the additional components into our model has not led to a change in the number of parameters. Consequently, the computational cost remains relatively unaffected by these enhancements. Therefore, since we are only using the smallest model, the number of parameters is fixed in 8.94 million.

Table 8 represents the evaluation metrics and their values for Case Study A.

By analyzing Table 8, it is possible to observe that the YOLOX-Ray configuration, which integrates both the SimAM attention mechanism and Alpha-*IoU* loss function, outperforms all other configurations in all metrics, except for *P* and inference time. Moreover, it achieved a high *R* value of 0.917, the highest $mAP_{50}$ value of 0.877 and the highest $mAP_{50:95}$ value of 0.422 when compared to other configurations. Nevertheless, its *p* value of 0.73 was slightly lower than the YOLOX configuration. In terms of speed, the YOLOX-Ray configuration boasted a relatively high FPS value of 83.68 and a low inference time of 11.95.

The YOLOX configuration secured the highest *p* value of 0.77, but possessed a $mAP_{50}$ value of 0.857 and an $mAP_{50:95}$ value of 0.40. It also demonstrated the highest FPS value of 84.89 and the lowest inference time of 11.78 ms, indicating rapid processing speed in this case study.

Regarding the other configurations with alternative attention mechanisms, YOLOX + SENet achieved a *p* value of 0.397, an *R* value of 0.891 and a $mAP_{50}$ value of 0.827. YOLOX + CBAM reported a *p* value of 0.431, an *R* value of 0.872 and a $mAP_{50}$ value of 0.797. Lastly, YOLOX + CA obtained a *p* value of 0.468, an *R* value of 0.888 and a $mAP_{50}$ value of 0.828. Among these, the YOLOX + CA configuration demonstrated the best performance in terms of *P* and *R*, while the YOLOX + SimAM configuration achieved the highest $mAP_{50}$ value in all attention mechanisms.

For the configurations with alternative loss functions, YOLOX + *CIoU* achieved a *p* value of 0.601, an *R* value of 0.913 and a $mAP_{50}$ value of 0.871. YOLOX + *DIoU* obtained a *p* value of 0.551, an *R* value of 0.9 and a $mAP_{50}$ value of 0.84. Lastly, YOLOX + *GIoU* reported a *p* value of 0.466, an *R* value of 0.885 and a $mAP_{50}$ value of 0.823. Among these, the YOLOX + *CIoU* configuration demonstrated the best performance in terms of *P*, *R* and $mAP_{50}$ values.

In conclusion, the ablation study results for Case Study A reveal that the YOLOX-Ray configuration delivered the best overall performance among all configurations, despite having a slightly lower *p* value than the YOLOX configuration. Such observations allow us to state that the combination of the SimAM attention mechanism and the Alpha-*IoU* loss function can effectively enhance the YOLOX-Ray architecture’s performance. However, the specific performance of each configuration depends on the task and dataset characteristics and the balance between speed and mAP must be considered when choosing the appropriate configuration.

Table 9 represents the evaluation metrics and their values for Case Study B.

Table 9 demonstrates that the YOLOX-Ray configuration achieved the highest values across all metrics, with the exception of inference time. The configuration obtained the highest $mAP_{50}$ value of 0.996, the highest $mAP_{50:95}$ value of 0.66, the highest *p* value of 0.984 and the highest *R* value of 0.987. However, it experienced a slightly higher inference time of 9.62 ms and a lower FPS value of 103.95 compared to the YOLOX base configuration.

Regarding the attention mechanisms, the YOLOX + SENet configuration achieved a *p* value of 0.699, an *R* value of 0.76, an $mAP_{50}$ value of 0.821 and an $mAP_{50:95}$ value of 0.357. The YOLOX + CBAM configuration reached a *p* value of 0.719, an *R* value of 0.88, an $mAP_{50}$ value of 0.845 and an $mAP_{50:95}$ value of 0.361. The YOLOX + CA configuration obtained a *p* value of 0.722, an *R* value of 0.961, an $mAP_{50}$ value of 0.963 and an $mAP_{50:95}$ value of 0.555. Among these, the YOLOX + SimAM configuration demonstrated the best performance in terms of *P*, *R* and mAP values.

For the configurations with alternative loss functions, the YOLOX + *CIoU* configuration achieved a *p* value of 0.933, an *R* value of 0.98, an $mAP_{50}$ value of 0.989 and an $mAP_{50:95}$ value of 0.61. The YOLOX + *DIoU* configuration obtained a *p* value of 0.913, an *R* value of 0.966, an $mAP_{50}$ value of 0.951 and an $mAP_{50:95}$ value of 0.581. The YOLOX + *GIoU* configuration reported a *p* value of 0.912, an *R* value of 0.964, an $mAP_{50}$ value of 0.911 and an $mAP_{50:95}$ value of 0.563. Among these, the YOLOX + Alpha-*IoU* configuration demonstrated the best performance in terms of *P*, *R* and mAP values.

In summary, the YOLOX-Ray configuration is the best choice for object detection in the crack detection case study, as it achieved the highest values in almost all metrics except inference time. The YOLOX base configuration is not recommended due to its poor performance in most metrics. While the addition of SimAM or Alpha-*IoU* improved certain metrics individually, the combination of both led to a better performance. It is important to note that lower inference times are preferred in real-time applications and higher FPS values signify the model’s capacity to process images more rapidly. Consequently, the YOLOX-Ray configuration demonstrated superior performance in terms of mAP while maintaining exceptional performance in terms of speed.

Table 10 represents the evaluation metrics and their values for Case Study C.

By analyzing Table 10, it becomes clear that the YOLOX-Ray configuration surpassed all other configurations regarding the two most challenging metrics, $mAP_{50}$ and $mAP_{50:95}$, obtaining values of 0.859 and 0.484, respectively. Nevertheless, it had a slightly lower *R* value of 0.866 compared to the YOLOX + SimAM configuration, suggesting that it failed to detect some true positive objects. This configuration also had a relatively low inference time of 18.04 ms and a relatively high FPS value of 55.43, making it slightly slower than the quickest configurations (YOLOX and YOLOX + SimAM).

The YOLOX base configuration exhibited the weakest performance in nearly all metrics, with a *p* value of 0.29, an *R* value of 0.821, an $mAP_{50}$ value of 0.768, an $mAP_{50:95}$ value of 0.389. However, it had the lowest inference time (17.47 ms) and consequently the highest FPS value (57.24). Curiously, despite being the simplest architecture, it achieved the same inference time as the YOLOX + SimAM configuration.

For the attention mechanisms, both the YOLOX + SimAM and YOLOX + CA configurations achieved lower values of $mAP_{50}$ and $mAP_{50:95}$ in comparison to the YOLOX-Ray configuration. Specifically, the YOLOX + SimAM configuration reached a slightly lower $mAP_{50}$ value of 0.84 and a lower $mAP_{50:95}$ value of 0.45 when compared to the YOLOX-Ray configuration, while the YOLOX + CA configuration secured a slightly higher $mAP_{50}$ value of 0.858 and a lower $mAP_{50:95}$ value of 0.439 when compared to the YOLOX-Ray configuration. Both configurations had high *R* values relative to YOLOX-Ray, with YOLOX + SimAM exhibiting the highest *R* value of 0.871 and YOLOX + CA displaying an *R* value of 0.866. Concerning inference time and FPS, YOLOX + SimAM had also the lowest inference time of 17.47 ms and, consequently, the highest FPS value of 57.24 among all configurations, while YOLOX + CA had a slightly longer inference time of 18.58 ms and a lower FPS value of 53.81.

Regarding the loss functions, the YOLOX + Alpha-*IoU* configuration achieved lower values of $mAP_{50}$ and $mAP_{50:95}$ in comparison to the YOLOX-Ray configuration, securing an even lower $mAP_{50}$ value of 0.806 and a lower $mAP_{50:95}$ value of 0.402. The YOLOX + Alpha-*IoU* configuration had a high *R* value relative to YOLOX-Ray, displaying an *R* value of 0.841. Concerning inference time and FPS, YOLOX + Alpha-*IoU* had a slightly longer inference time of 18.20 ms and a lower FPS value of 54.95.

In summary, the YOLOX-Ray configuration delivered the best performance in terms of the most crucial metrics ($mAP_{50}$ and $mAP_{50:95}$), despite having a lower *R* value and slower inference times compared to some other configurations. The results also imply that, in this case study, faster inference times and higher FPS values are preferable but should not undermine model performance. The attention mechanisms and loss functions individually showed improvements over the base YOLOX configuration, but the combination of these techniques in the YOLOX-Ray configuration led to the most significant performance gains.

## 5. Conclusions

The experimental results presented in this study demonstrate the YOLOX-Ray deep learning architecture’s effectiveness in the context of industrial inspection tasks. We demonstrated the YOLOX-Ray model’s versatility in a wide range of scenarios by evaluating its performance across three distinct case studies of variable complexity.

The ablation study’s findings revealed that incorporating SimAM and Alpha-*IoU* individually improved the performance of the YOLOX base architecture. However, when combining both components (YOLOX-Ray) the best overall results can be achieved. These findings highlight the importance of selecting and fine-tuning the various components of deep learning architectures for specific use cases. They can also serve as a foundation for future industrial inspection research and development.

The YOLOX-Ray architecture improves object detection in real-world industrial inspection scenarios by incorporating both the SimAM attention mechanism and the Alpha-*IoU* loss function. By incorporating the SimAM module, the architecture can effectively refine features in both the channel and spatial domains, resulting in a more accurate and robust object detection performance. SimAM’s lightweight and efficient design allows for increased computational efficiency while maintaining detection quality. The Alpha-*IoU* loss function, on the other hand, addresses the challenges of detecting small objects and scale variations, making it suitable for difficult industrial inspections and anomaly detection tasks.

Due to the combination of the SimAM attention mechanism and the Alpha-*IoU* loss function, the YOLOX-Ray architecture performs better, in particular, on complex and challenging environments, ensuring more precise detections and improved overall performance. The YOLOX-Ray architecture is an optimal choice for tackling real-world object detection tasks and applications due to its powerful fusion of attention mechanism and loss function.

## Figures and Tables

**Table 1:** Alpha-*IoU* vs. CIoU loss functions in terms of mAP. (Source: [38]).

Loss Function	Object Scale	Dataset	*mAP*
CIoU	Single-scale	DOTA-v1.0	0.7709
		DOTA-v1.5	0.7287
	Multi-scale	DOTA-v1.0	0.78
		DOTA-v1.5	0.7502
Alpha-*IoU*	Single-scale	DOTA-v1.0	0.7761
		DOTA-v1.5	0.7333
	Multi-scale	DOTA-v1.0	0.7877
		DOTA-v1.5	0.7506

**Table 2:** Technical details of the datasets for each case study.

	Images	Train Set	Validation Set	Test Set	Classes
Case Study A	1200	840	240	120	Fault
Case Study B	2144	1500	433	211	crack
Case Study C	880	616	176	88	slippage corrosion crack

**Table 3:** YOLOX-Ray network hyperparameters.

Hyperparameter	Value
Epochs	300
Activation Function	SiLU
Optimizer	RADAM
Initial LR	0.05
LR Scheduler	Cosine Annealing
Data Augmentations	MOSAIC, MixUP, HSV, Flip H/V
Alpha-*IoU* (α)	3

**Table 4:** Depth and width values for each YOLOX-Ray model size.

	Small (s)	Medium (m)	Large (l)	Extra-Large (x)
Depth	0.33	0.67	1.0	1.33
Width	0.50	0.75	1.0	1.25

**Table 5:** Performance evaluation of the models on Case Study A.

Model	*P*	*R*	*mAP* ${}_{50}$	*mAP* ${}_{50:95}$	*Inf.* (ms)	Params ($10^{6}$)
YOLOX-Ray-s	0.73	0.917	0.877	0.422	11.95	8.94
YOLOX-Ray-m	0.829	0.915	0.872	0.426	19.55	25.28
YOLOX-Ray-l	0.806	0.916	0.89	0.427	29.22	54.15
YOLOX-Ray-x	0.733	0.879	0.845	0.376	46.56	99.0

**Table 6:** Performance evaluation of the models on Case Study B.

Model	*P*	*R*	*mAP* ${}_{50}$	*mAP* ${}_{50:95}$	*Inf.* (ms)	Params ($10^{6}$)
YOLOX-Ray-s	0.984	0.987	0.996	0.66	9.62	8.94
YOLOX-Ray-m	0.972	0.975	0.994	0.661	17.09	25.28
YOLOX-Ray-l	0.962	0.979	0.994	0.658	25.96	54.15
YOLOX-Ray-x	0.972	0.971	0.977	0.625	42.53	99.0

**Table 7:** Performance evaluation of the models on Case Study C.

Model	*P*	*R*	*mAP* ${}_{50}$	*mAP* ${}_{50:95}$	*Inf.* (ms)	Params ($10^{6}$)
YOLOX-Ray-s	0.762	0.866	0.859	0.484	18.04	8.94
YOLOX-Ray-m	0.829	0.878	0.871	0.499	26.60	25.28
YOLOX-Ray-l	0.792	0.883	0.873	0.505	37.83	54.15
YOLOX-Ray-x	0.832	0.876	0.877	0.518	58.12	99.0

**Table 8:** Ablation study results for Case Study A.

Configuration	*P*	*R*	*mAP* ${}_{50}$	*mAP* ${}_{50:95}$	*Inf.* (ms)	FPS
YOLOX	0.77	0.91	0.857	0.40	11.78	84.89
Attention Mechanisms						
YOLOX + SENet	0.397	0.891	0.827	0.332	12.14	82.37
YOLOX + CBAM	0.431	0.872	0.797	0.315	12.46	80.26
YOLOX + CA	0.468	0.888	0.828	0.324	12.05	82.99
YOLOX + SimAM	0.359	0.916	0.861	0.371	12.32	81.17
Loss Functions						
YOLOX + *CIoU*	0.601	0.913	0.871	0.378	11.89	84.10
YOLOX + *DIoU*	0.551	0.9	0.84	0.34	12.44	80.38
YOLOX + *GIoU*	0.466	0.885	0.823	0.315	11.97	83.54
YOLOX + Alpha-*IoU*	0.464	0.915	0.866	0.378	12.18	82.10
Proposed Method						
YOLOX-Ray	0.73	0.917	0.877	0.422	11.95	83.68

**Table 9:** Ablation study results for Case Study B.

Configuration	*P*	*R*	*mAP* ${}_{50}$	*mAP* ${}_{50:95}$	*Inf.* (ms)	FPS
YOLOX	0.67	0.931	0.897	0.33	9.59	104.28
Attention Mechanisms						
YOLOX + SENet	0.699	0.76	0.821	0.357	10.45	95.69
YOLOX + CBAM	0.719	0.88	0.845	0.361	9.98	100.2
YOLOX + CA	0.722	0.961	0.963	0.555	9.7	103.09
YOLOX + SimAM	0.97	0.986	0.99	0.634	9.71	102.99
Loss Functions						
YOLOX + *CIoU*	0.933	0.98	0.989	0.61	9.72	102.88
YOLOX + *DIoU*	0.913	0.966	0.951	0.581	9.81	101.94
YOLOX + *GIoU*	0.912	0.964	0.911	0.563	9.86	101.41
YOLOX + Alpha-*IoU*	0.957	0.972	0.976	0.569	9.91	100.91
Proposed Method						
YOLOX-Ray	0.984	0.987	0.996	0.66	9.62	103.95

**Table 10:** Ablation study results for Case Study C.

Configuration	*P*	*R*	*mAP* ${}_{50}$	*mAP* ${}_{50:95}$	*Inf.* (ms)	FPS
YOLOX	0.29	0.821	0.768	0.389	17.47	57.24
Attention Mechanisms						
YOLOX + SENet	0.585	0.87	0.856	0.445	17.90	55.87
YOLOX + CBAM	0.577	0.861	0.849	0.422	18.50	54.05
YOLOX + CA	0.521	0.866	0.858	0.439	18.58	53.81
YOLOX + SimAM	0.277	0.871	0.84	0.45	17.47	57.24
Loss Functions						
YOLOX + *CIoU*	0.657	0.857	0.846	0.435	18.33	54.56
YOLOX + *DIoU*	0.649	0.857	0.846	0.437	18.16	55.07
YOLOX + *GIoU*	0.604	0.846	0.825	0.427	17.64	56.69
YOLOX + Alpha-*IoU*	0.304	0.841	0.806	0.402	18.20	54.95
Proposed Method						
YOLOX-Ray	0.762	0.866	0.859	0.484	18.04	55.43

## Data Availability

The YOLOX-Ray source code, as well as the model’s weights for pre-training other custom datasets can be found on this https://github.com/Sphincz/YOLOX-Ray (GitHub repository). The datasets used in the case studies can be found in the https://www.rf100.org/ (Roboflow-100) official website.

## Abbreviations

The following abbreviations are used in this manuscript:

AI	Artificial Intelligence
AP	Average Precision
CA	Coordinate-Attention
CAM	Channel Attention Mechanism
CBAM	Convolutional Block Attention Module
CCA	Coordinate-Channel Attention
CE	Cross-Entropy
*CIoU*	Complete Intersection-over-Union
CNN	Convolutional Neural Network
COCO	Common Objects in Context
CPU	Central Process Unit
CSA	Coordinate-Spatial Attention
CSP	Cross Stage Partial
CV	Computer Vision
*DIoU*	Distance Intersection-over-Union
DL	Deep Learning
DNN	Deep Neural Network
DOTA	Dataset for Object Detection in Aerial images
FPN	Feature Pyramid Network
FPS	Frames Per Second
*GIoU*	Generalized Intersection-over-Union
GPU	Graphics Processing Unit
*GT*	Ground-Truth
HSV	Hue, Saturation and Value
*IoU*	Intersection-over-Union
mAP	Mean Average Precision
ML	Machine Learning
OTA	Optimal Transport Assignment
PAN	Path Aggregation Network
RPN	Region Proposal Network
SAM	Spatial Attention Mechanism
SE	Squeeze-and-Excitation
SENet	Squeeze-and-Excitation Network
SimAM	Simple Parameter-free Attention Module
SimOTA	Simplified Optimal Transport Assignment
SoTA	State-of-the-Art
VOC	Visual Object Classes
YOLO	You Only Look Once
